# Language context modulates reading route: an electrical neuroimaging study

**DOI:** 10.3389/fnhum.2014.00083

**Published:** 2014-02-20

**Authors:** Karin A. Buetler, Diego de León Rodríguez, Marina Laganaro, René Müri, Lucas Spierer, Jean-Marie Annoni

**Affiliations:** ^1^Neurology Unit, Laboratory for Cognitive and Neurological Sciences, Department of Medicine, Faculty of Science, University of FribourgFribourg, Switzerland; ^2^Faculty of Psychology and Educational Sciences, University of GenevaGeneva, Switzerland; ^3^Division of Cognitive and Restorative Neurology, Departments of Neurology and Clinical Research, Inselspital, University Hospital, University of BernBern, Switzerland

**Keywords:** reading, pseudoword, orthographic depth, grapheme-phoneme conversion, dual-route model, EEG, ERP, bilingual

## Abstract

**Introduction:** The orthographic depth hypothesis (Katz and Feldman, 1983) posits that different reading routes are engaged depending on the type of grapheme/phoneme correspondence of the language being read. Shallow orthographies with consistent grapheme/phoneme correspondences favor encoding via non-lexical pathways, where each grapheme is sequentially mapped to its corresponding phoneme. In contrast, deep orthographies with inconsistent grapheme/phoneme correspondences favor lexical pathways, where phonemes are retrieved from specialized memory structures. This hypothesis, however, lacks compelling empirical support. The aim of the present study was to investigate the impact of orthographic depth on reading route selection using a within-subject design.

**Method:** We presented the same pseudowords (PWs) to highly proficient bilinguals and manipulated the orthographic depth of PW reading by embedding them among two separated German or French language contexts, implicating respectively, shallow or deep orthography. High density electroencephalography was recorded during the task.

**Results:** The topography of the ERPs to identical PWs differed 300–360 ms post-stimulus onset when the PWs were read in different orthographic depth context, indicating distinct brain networks engaged in reading during this time window. The brain sources underlying these topographic effects were located within left inferior frontal (German > French), parietal (French > German) and cingular areas (German > French).

**Conclusion:** Reading in a shallow context favors non-lexical pathways, reflected in a stronger engagement of frontal phonological areas in the shallow versus the deep orthographic context. In contrast, reading PW in a deep orthographic context recruits less routine non-lexical pathways, reflected in a stronger engagement of visuo-attentional parietal areas in the deep versus shallow orthographic context. These collective results support a modulation of reading route by orthographic depth.

## INTRODUCTION

Grapheme to phoneme conversion is a critical step in reading processing, as notably evidenced by its role in literacy acquisition (Goswami, 1998; Seymour et al., 2003; Huang et al., 2004; Lallier et al., 2013) and, when impaired, in the emergence of language-related disorders including dyslexia (Goswami, 1998; Wheat et al., 2010). Referred to as orthographic regularity, the rules of grapheme to phoneme conversion vary considerably across stimuli and languages. Consequently, reading strategies must be adjusted depending on the writing systems involved (in terms of orthographic transparency-opacity; Katz and Feldman, 1983). However, how reading strategies and the underlying brain network are actually modified when reading languages with different orthographic regularities remains largely unresolved.

According to the dual route cascade model (Coltheart et al., 2001; for a review see Jobard et al., 2003), after letter identification, word reading processing may follow two pathways mapping differently graphemes to phonemes. On the non-lexical pathway, each grapheme is sequentially mapped to its corresponding phoneme (grapho-phonological assembling). The non-lexical route has been advanced to be predominantly involved when reading unfamiliar words or letter strings (non-words; Coltheart et al., 2001; Proverbio and Zani, 2003; Proverbio et al., 2004; Heim et al., 2005; Lu et al., 2011). In contrast, the reading of familiar words may preferentially involve the faster lexical pathways, where phonemes are retrieved from memory structures, i.e., from orthographic and phonological lexical entries (lexico-semantic access; Coltheart et al., 2001; Proverbio and Zani, 2003; Proverbio et al., 2004; Lu et al., 2011; Fisher et al., 2012).

Studies identifying the neural correlates of the two routes by contrasting word versus pseudoword (PW) reading yielded diverging results. In reading tasks, greater activation for PWs than words was found in both, left occipito-temporal and inferior frontal regions (Xu et al., 2001; Mechelli et al., 2003; Kronbichler et al., 2004; Binder et al., 2005). In contrast, studies using lexical decision tasks have reported greater activation for words than PWs in left occipito-temporal cortices, along with stronger or equivalent activation to PWs in left inferior frontal regions (Fiebach et al., 2002; Rissman et al., 2003; Ischebeck et al., 2004; Binder et al., 2005). Finally, evidence has been found for an equal engagement of occipito-temporal regions in early word and PW reading (Jobard et al., 2003; Wilson et al., 2007). In a review of 35 neuroimaging studies, Jobard et al. (2003) suggest that pre-lexical processing does not differentiate between words and PWs and the selection in favor of one route occurs at later stages. On the non-lexical route, after pre-lexical processing, regular words and PWs are encoded via grapho-phonological conversion. The grapho-phonological route relies on left superior temporal, supramarginal, and inferior frontal areas (pars opercularis; BA 44). On the lexical route, after pre-lexical processing, regular and irregular words are encoded via lexico-semantic representations. These lexico-semantic areas involve basal inferior and posterior middle temporal and inferior frontal areas (pars triangularis; BA 45).

More recent studies confirmed these results by linking the lexical processing of words to bilateral posterior cingular, inferior-middle temporal and temporo-parietal regions (Ischebeck et al., 2004). In contrast, left posterior superior temporal (Graves et al., 2008), supramarginal (Roux et al., 2012) and inferior frontal regions (Ischebeck et al., 2004; Nixon et al., 2004; Heim et al., 2005; Rodriguez-Fornells et al., 2006; Wheat et al., 2010) were identified to subserve non-lexical word and PW processing.

In addition, lesion-based studies of acquired dyslexia found that surface dyslexia, where due to impaired lexical pathways patients are able to read pronounceable PWs and unable to read (irregular) words, goes in line with deficits in inferior temporal (Mechelli et al., 2005; Price and Mechelli, 2005), anterior temporal (Woollams et al., 2007) and anterior inferior frontal regions (Mechelli et al., 2005). In contrast, phonological dyslexia with impaired non-lexical pathways, where patients are able to read most high-frequency words (i.e., regular and irregular) and unable to process simple PWs, has been linked to deficits in left inferior-parietal (Rapcsak et al., 2009) and left inferior frontal regions (Feiz et al., 2006).

The inconsistent findings related to the anatomical underpinnings of reading routes may be due to differences in tasks applied, ranging from reading paradigms (Mechelli et al., 2003; Kronbichler et al., 2004; Binder et al., 2005) to lexical decision (Fiebach et al., 2002; Rissman et al., 2003; Ischebeck et al., 2004; Binder et al., 2005; Wilson et al., 2007) and rhyming tasks (Xu et al., 2001). In addition, the results may be impacted by differences of the stimuli used (especially differences in the lexicality of PWs). Finally, differences in the orthography of the language investigated may have influenced the findings, as some studies investigated languages with regular (Fiebach et al., 2002; Ischebeck et al., 2004; Kronbichler et al., 2004) and irregular (Xu et al., 2001; Mechelli et al., 2003; Rissman et al., 2003; Binder et al., 2005; Wilson et al., 2007; Wheat et al., 2010) orthographies.

In addition to the degree of the lexicality or familiarity of the words being read, the orthographic depth hypothesis (Katz and Feldman, 1983; Katz and Frost, 1992) posits that the differential engagement of each reading pathway depends on the transparency of the language’s grapheme to phoneme correspondence. Shallow orthographies (e.g., German and Italian) with consistent grapheme to phoneme correspondences favor encoding via non-lexical pathways (assembled reading strategy), whereas deep orthographies (e.g., French and English), with an inconsistent grapheme to phoneme correspondence favor lexical pathways (addressed reading strategy).

Of note, the engagement of a given pathway is not exclusive, i.e., reading processing generally involves both routes, but one route may be predominantly activated compared to the other depending on the orthographic depth index of the language (Heim et al., 2005; Mousikou et al., 2010; Timmer et al., 2012).

Only few studies have brought evidence for a modulation of brain activity during reading by orthographic depth of the used language. In a PET study contrasting English and Italian monolinguals, Paulesu et al. (2000) observed that English readers showed stronger activations than Italian readers within areas suggested to be involved in irregular word reading (left posterior inferior temporal and anterior inferior frontal). By contrast, monolingual Italian readers showed stronger activity than English readers in areas involved in phonological transcoding processing (left superior temporal). Simon et al. (2006) further showed on French monolinguals and French-Arabic bilinguals (with Arabic being the deeper orthography) that the N320 electroencephalography (EEG) component differentiated reading French words and PWs and Arabic words. The 300 ms latency has been associated with orthographic-linguistic processing, especially spelling-to-sound conversion (Bentin et al., 1999; Huang et al., 2004; Proverbio et al., 2004; Simon et al., 2004, 2006; Grainger et al., 2006; Hauk et al., 2006; Ashby et al., 2009; Carreiras et al., 2009). Similarly, in a study examining Hebrew bilinguals with a shallow and deep version of Hebrew script, Bar-Kochva and Breznitz (2012) showed larger event-related potential (ERP) amplitudes to the deep script 340 ms after word onset. Using the same paradigm with Hebrew bilinguals, Frost (1994) showed larger word frequency and semantic priming effects when reading words written in deep compared to shallow script. The author interpreted their results in terms of facilitated semantic access due to predominant engagement of lexical pathways.

Of note, relative early latencies have also been found to be critically engaged in graphemic/phonologic conversion. Wheat et al. (2010) found neurophysiological correlates to phonological processing starting as early as 100 ms after word onset in English readers. Proverbio and Zani (2003) and Sereno et al. (1998) found differences at 160 ms after word onset to support grapheme to phoneme conversion in Italian and English readers, respectively. In contrast, relatively late latencies were found in a rhyme task conducted by Rugg (1984), Rugg and Barrett (1987), suggesting the N450 to be critical for phonological processing. However, studies evidencing for an early (<200 ms; Sereno et al., 1998; Proverbio and Zani, 2003; Wheat et al., 2010), resp. late (>400ms; Rugg, 1984; Rugg and Barrett, 1987) grapho-phonological processing did not directly manipulate the impact of orthographic depth on grapheme to phoneme conversion. In contrast, studies explicitly manipulating the effect of orthographic depth consistently report latencies around 300 ms (Simon et al., 2006; Bar-Kochva and Breznitz, 2012) to be critically engaged in grapheme to phoneme mapping.

Collectively, the results indicate that a modulation of orthographic depth may impact reading routes around 300 ms after stimulus onset. Grapheme to phoneme mapping in languages with shallow orthographies seems to rely on regions involved in grapho-phonological processing (superior temporal, supramarginal and opercular inferior frontal regions), indicating an activation of non-lexical pathways. In contrast, grapheme to phoneme mapping in languages with deep orthographies seem to rely on regions involved in lexico-semantic processing (inferior and middle temporal and triangular inferior frontal regions), indicating an activation of lexical pathways. Thus, orthographic depth may indeed impact reading route selection.

However, because previous studies used between-subject or cross-language designs, the conclusions about the effect of orthographic depth that can be drawn from current literature are limited. In between-subject designs (Paulesu et al., 2000), inter-subject heterogeneity resulting from a variety of socio-cultural differences may indeed account for the observed effects. For example, the differences found in the cited studies may reflect different reading habits, differences in education or intelligence across groups rather than differences in orthographic processing across languages. One way of minimizing confounds arising from inter-subject comparisons is to investigate reading in bilingual subjects. Since there is evidence for a certain degree of independency in word processing for each language (Soares and Grosjean, 1984; Rodriguez-Fornells et al., 2006; Kovelman et al., 2008), bilingualism is an advantageous model to investigate reading strategies. Bilinguals, particularly natural bilinguals, have the possibility to engage in several language modes independently, i.e., to adapt to the specific linguistic constraints of a language (Soares and Grosjean, 1984; Rodriguez-Fornells et al., 2006). A bilingual reader, being native in a shallow and a deep language, should thus be able to apply an assembled strategy when reading the shallow orthography and an addressed strategy when reading the deep orthography. However, so far studies on reading strategies in bilinguals applied cross-language designs (Simon et al., 2006) or altered scripts within one language (Frost, 1994; Bar-Kochva and Breznitz, 2012). In cross-language designs, the effect of orthographic depth may be confounded with effects resulting from comparison across different linguistic stimuli. The same holds for comparisons between different stimuli within one language, as the impact of orthographic depth may be confounded with physical differences between the visual stimuli.

The aim of the present study was to investigate the impact of orthographic depth on reading route selection using an experimental design excluding possible effects related to differences in stimuli or readers. We presented the same PWs to highly proficient bilinguals and manipulated the orthographic depth of PW reading by embedding them among two separated language contexts respectively implicating shallow or deep orthography. The use of PWs as target stimuli will probably strengthen non-lexical processing independent of language contexts. In contrast, the reading route predominantly engaged during a language context will depend on its orthographic depth: the deep language context may strengthen lexical and the shallow language context non-lexical pathways. Consequently, if orthographic depth modulates reading routes, reading in the shallow context will support the non-lexical pathways routinely recruited to process PWs. In contrast, non-lexical pathways routinely recruited to process PWs may be less engaged when reading in the deep compared to the shallow context. Together, we predict a differential engagement of non-lexical pathways between pre-lexical and semantic processing stages (~300 ms) reflected in a stronger activation of grapho-phonological (superior temporal, supramarginal and inferior frontal) areas in the shallow versus the deep language context when reading identical PWs across language context. The study of early/high proficient bilinguals enabled controlling for socio-cultural effects and the use of identical stimuli across conditions for effects due to linguistic and/or physical differences, thus isolating the effect of orthographic depth in the 1 versus 1 within-subject design.

## MATERIALS AND METHODS

### PARTICIPANTS

Fourteen healthy female French/German bilinguals participated in the study (all right-handed, Oldfield, 1971), aged 18–24 years (mean = 20.86 years, SD = 2.03 years). All participants learnt French and German before the age of six and showed balanced high proficiency across languages according the bilingual questionnaire they filled out (**Table 1**, see “Language evaluation”). No participant had a history of reading difficulties, neurological or psychiatric illness and all reported normal or corrected-to-normal vision. Each participant provided written, informed consent to participate in the study. The study was approved by the Ethics Committee of the University of Fribourg.

**Table 1:** French-German bilingualism characteristics of participants (*N* = 14).

Variable	French		German		*p* value (uncorrected)
	mean	SD	mean	SD	
Age of acquisition (years)	1.61	2.21	0.93	1.87	0.43
Lived in region speaking (years)	16.65	6.33	11.92	9.34	0.19
**Family (%)**
First language mother	14	35	79	41	0.01*
Language spoken with mother	50	50	57	49	0.78
First language father	50	50	50	50	1.00
Language spoken with father	58	49	46	50	0.55
**Childhood (Age < 7 years) (%)**
Language taught in school	48	43	52	43	0.88
Language spoken with peers at school	52	44	41	43	0.65
Language spoken with family	45	32	45	32	1.00
**Present (%)**
Spoken at work	52	26	48	26	0.81
Watching TV/listening radio	30	22	66	24	0.01*
Speaking with friends	59	22	38	21	0.09
Reading books	43	24	54	26	0.45
Mental arithmetic	64	35	27	31	0.04*
**Learned (%)**
Learned in school only	7	26	0	0	-
Learned “on road” only	0	0	0	0	-
Learned at workplace only	0	0	0	0	-
**Self-evaluation (%)**
Speaking	94	6	92	11	0.57
Comprehension	97	4	96	5	0.82
Reading	90	11	90	12	0.91
Writing	77	22	80	17	0.64
**Computer-based evaluation (min = 0; max = 1000)**	
DIALANG score	852	124	864	59	0.77

SD, standard deviation; *p < 0.05.

### LANGUAGE EVALUATION

All participants filled out a questionnaire evaluating French and German language skills consisting of three parts (**Table 1**): immersion, self-evaluation and computer-based evaluation. To asses language immersion, participants were asked for the age of acquisition, how long they lived in a region where predominantly German or French was spoken, which language they spoke with family members, during their childhood, in present activities, and if the language was acquired in school or out of school only. For the self-evaluation part, participants had to indicate in percentages how well they would estimate their reading, speaking, comprehension and writing skills. Finally, a sub-test from the computer-based DIALANG language diagnosis system (Zhang and Thompson, 2004) was performed to evaluate reading performance. Here, the task was to indicate for each of 75 stimuli whether it was a correct word in the corresponding language or a (highly word-like) PW. The score ranged between 0 and 1000, with a score >900 being mother tongue (L1) level and a score from 601 to 900 being fully functional with little to no difficulty in reading.

### STIMULI

Target stimuli of the study were orthotactic (i.e., orthographically legal) PWs composed of 4 to 6 letters (to avoid eye movements). One hundred and twenty PWs were generated using WordGen software (Duyck et al., 2004) and matched for their lexical distance to French and German language respecting summated bigram frequency [the frequency of all adjacent letter pairs of an item: e.g., for the item “word” the frequencies of the bigrams “wo,” “or” and “rd” were summed up; French mean = 14005, German mean = 13773; *t*(119) = 0.631, *p* = 0.53; WordGen, Duyck et al., 2004], neighborhood size [the number of existing words that can be obtained by changing one letter of the item; French mean = 1.57, German mean = 1.45; *t*(119) = 0.781, *p* = 0.44; WordGen, Duyck et al., 2004], bi- and tri-gram legality (Lexique, New et al., 2001; lexikalische Datenbank; lexical database (dlexDB), Digitales Wörterbuch der deutschen Sprache; Digital Dictionary of the German Language (DWDS), Geyken, 2007), onset phoneme legality (PWs started with a phoneme frequently used as a first phoneme in real words; Lexique, New et al., 2001; dlexDB, DWDS, Geyken, 2007) and letter (position independent and onset letter) frequency, which was fitted to the letter frequency distribution of each language (Best, 2005; CorpusDeThomasTempé, retrieved May 2012). Examples of PWs include: *Nate, Dand, Melle, Apase, Gantel*, and *Grutte*.

French and German words were presented in addition to the PWs to strengthen language context (see “Procedure and Task”). Four hundred and eighty French Words were selected from Lexique database (New et al., 2001) and 480 German Words were selected from CELEX database (Baayen et al., 1995). Words were closely matched across languages on length [WordGen, Duyck et al., 2004; French mean = 5 letters, German mean = 5 letters; *t*(958) = 0.000, *p* = 1.000], log-transformed lexical frequency [French mean = 1.59, German mean = 1.60; *t*(958) = 0.250, *p* = 0.803], neighborhood size [French mean = 3.31, German mean = 3.31; *t*(958) = 0.000, *p* = 1.000], summated bigram frequency [French mean = 11328, German mean = 11447; *t*(958) = 0.312, *p* = 0.755] and length in syllables [French mean = 1.52, German mean = 1.59; *t*(958) = 1.856, *p* = 0.064]. Examples of words include: *Noël, Trou, Année, Maman, Violon*, and *Esprit* (French) and *Maus, Kind, Draht, Seite, Prämie*, and *Lösung* (German).

One hundred and twenty symbol strings (symbols) were created by changing the font of the PWs to “symbols” in MS Word (Microsoft Corporation, 2010). symbols were intended to be part of future research and were not analyzed in the present study. Examples of symbols are: Nατε, Δαυδ, Mελλε, Aπασε, Γαυτελ, Γρυττε.

### PROCEDURE AND TASK

The task in this study was to read aloud French and German words, PWs and symbols displayed on a computer screen.

**FIGURE 1:**
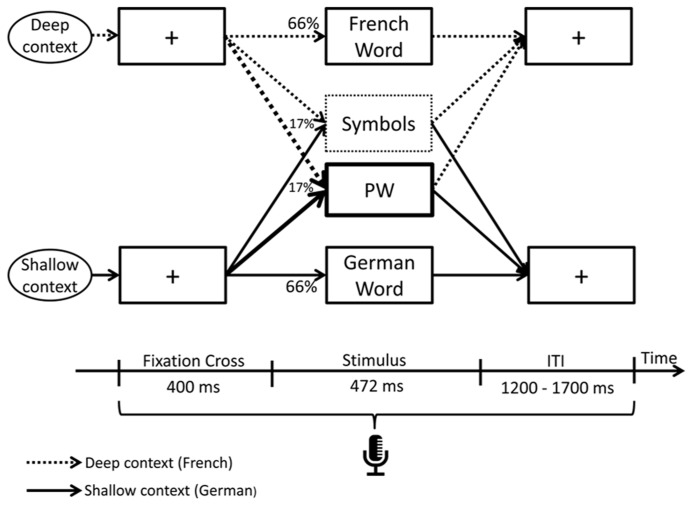
**Experimental paradigm.** Each trial started with the presentation of a fixation cross of 400 ms duration, followed by a pseudo-randomly determined stimulus (66% word, 17% pseudoword, or 17% symbols) displayed for 472 ms and terminated with a response window displaying a fixation cross with a random duration between 1200 and 1700 ms. Production latencies were recorded throughout each trial. Target stimuli of the study were pseudowords (PWs). To manipulate the orthographic depth of reading, the same PWs were embedded among two separated language context sessions: in the deep orthographic context, the words consisted of French and in the shallow orthographic context of German words. The order of language context sessions was randomized across participants.

Participants were seated in an electrically shielded and sound attenuated booth 90 cm in front of a 21-inch LCD screen. Stimulus delivery and response recording were controlled using E-Prime 2.0 (Psychology Tools, Inc., Pittsburgh, PA, USA). Stimuli were presented in the center of the screen and displayed in black font color on white background. Each trial started with the presentation of a fixation cross of 400 ms duration, followed by a pseudo-randomly determined stimulus (66% word, 17% PW or 17% symbol, see next paragraph) displayed for 472 ms to allow comfortable reading (Courier New, pt. 24). A response window displaying a fixation cross with a random duration between 1200 and 1700 ms was presented after the stimuli (inter trial interval; **Figure 1**).

Since the aim of the study was to investigate the effect of orthographic transparency on reading identical stimuli, orthographic depth of PW reading was manipulated by creating two separated language context sessions (experimental phase; **Figure 1**). To strengthen French language context (deep orthography), the 120 PWs (and 120 symbols) were embedded among 480 French words. In the French language context session, participants were asked to pronounce the PWs as if they were existing French words. To strengthen German language context (shallow orthography), the same 120 PWs (and 120 symbols) were embedded among 480 German words. In the German language context session, participants were asked to pronounce the PWs as if they were existing German words. The same procedure applied for the symbols, except that here, participants should try to recognize the symbols as lexical letter strings and pronounce them as if they were existing words in the given language (e.g., the symbol “μιϖoτε” could be read as “miwote”). E-Prime voice key was used to record audio responses and production latencies.

At the beginning of each language context session, a short text written in the corresponding language was presented in order to activate the given language. Next, a 2 min training block with words (not included in experimental phase) in the language of the selected context session was started to familiarize the procedure and verify the apparatus, before initiating the experimental phase. To reduce fatigue, stimuli presentation of one language context session was divided into four blocks separated by 1–2 min breaks. One block comprised of randomly selected 30 PWs, 30 symbols, and 120 words and lasted around 6 min. The order of blocks was randomized across participants. Both language context sessions were separated by a pause of at least 10 min. The order of language context sessions was randomized across participants.

### EEG ACQUISITION AND PREPROCESSING

Continuous EEG was acquired at 1024 Hz through a 128-channel Biosemi ActiveTwo system (Biosemi, Amsterdam, Netherlands) referenced online to the CMS-DRL ground, which functions as a feedback loop driving the average potential across the montage as close as possible to the amplifier zero. Electrode impedances were kept below 20 kOhm. EEG data preprocessing and analyses were conducted offline using Cartool (Brunet et al., 2011). EEG epochs from 100 ms pre-stimulus to 500 ms post-stimulus onset (i.e., 102 data points before and 512 data points after stimulus onset) were averaged and ERPs were calculated for each participant and condition (PW in French context versus PW in German context). symbols were excluded from analyses of the present study as they were intended to be the focus of future research. EEG epochs containing eye blinks or other noise transients were removed after visual inspection in addition to a ± 80 μV artifact rejection criterion at any channel. Data were band-pass filtered (0.18–40 Hz), notch filtered at 50 Hz and recalculated against the average reference. By removing slow drifts at the single epoch level, the high-pass filter resulted in a baseline correction on the whole epoch. Before group averaging, data at artifact electrodes from each participant were interpolated using a 3-dimensional spline algorithm (Mean 6.25% interpolated electrodes; Perrin et al., 1987). The average number ( ± SEM) of accepted epochs was 109 ± 2.90 for PWs in French context and 110 ± 2.11 for PWs in German context. These values did not differ statistically [*t*(13) = 0.377, *p* = 0.712], ruling out that our effects result from differences in signal-to-noise ratios across conditions.

### STATISTICAL ANALYSES

#### Behavioral analysis

Response accuracy of PWs and words was assessed by auditory inspection of the audio files generated with E-Prime to determine whether different language contexts were created successfully. Expected pronunciations were a priori defined by a native German and a native French speaker. Five types of errors were defined: language intrusion (complete or partial German pronunciation in French context or complete or partial French pronunciation in German context), orthography (adding, exchanging or omitting letters), phonology (unusual phonological coding of correct orthographic form), intonation (wrong lexical stressing), and other errors leading to an incorrect response (e.g., abortion, correction, no response, pronunciation in a third language).

Examples of language intrusions demonstrated on the PWs “nate” (correct response German = [’na:tə]; correct response French = [nat]), “melle” (correct response German = [’mεlə]; correct response French = [mεl]) and “apsase” (correct response German = [a’pa:zə]; correct response French = [’apa:z]) are: [’na:tə] resp. (’mεlə] (both complete) or [‘apa:zə] (partial) in French context and [nat] resp. [mεl] (both complete) or [a’pa:z] (partial) in German context. Examples for orthographic errors demonstrated on the PW “grutte” (correct response German = [’grυtə]; correct response French = [gRyt]) are: [’gυtə] or [’gυrtə] in German and [gyt] or [gyrt] in French. Examples for phonological errors demonstrated on the PW “dand” (correct response German = [dant]; correct response French = [dã]) are: [tand] in German context and [dãd] in French context. Examples for intonation errors demonstrated on the PW “gantel” (correct response German = [’gantl]; correct response French = [’gãtεl]) are: [gan’tel] in German context and [gã’tεl] in French context. Phonetic notations are represented according to the International Phonetic Alphabet (IPA; International Phonetic Association).

To investigate whether response accuracy rates differentiate or interact across conditions, a 2 × 2 repeated-measures analysis of variance (ANOVA) with factors language context (French vs. German) and Stimulus Type (Words vs. PW) was performed. In addition, a paired *t*-test was performed contrasting PWs in French context versus PWs in German context.

To investigate whether error types in PW reading differentiate across language contexts, a one-way repeated-measures multivariate analysis of variance (MANOVA) was performed. language context (French, German) was included into the analysis as independent and Language Intrusion Errors, Orthographic Errors and Phonological Errors in PW reading were included as dependent variables. Due to low incidences (see first paragraph of “Behavioral Results”), intonation errors and errors labeled as “other” were excluded from the analysis to increase statistical power. A series of one-way univariate analyses were performed as *post hoc* tests. To counteract alpha inflation due to multiple hypotheses testing in univariate analyses, Bonferroni correction was applied and significance threshold set at *p* < 0.02 (Dunn, 1961).

Production latencies [reaction times (RT)] were assessed with a speech analysis software (Praat; Boersma and Weenink, 2013) and compared across language context and Stimulus Type to determine whether they varied with the manipulated factors. Twelve participants were included into behavioral analyses and two had to be excluded due to invalid recordings. Trials containing RTs exceeding ± 2 standard deviations (SD) from the mean were considered as outliers/errors and excluded from analysis, which resulted in the removal of a total of 3% of trials from French context condition (mean number of excluded words = 15; mean number of excluded PWs = 4) and 3% of trials from German context condition (mean number of excluded words = 14; mean number of excluded PWs = 3).

To investigate whether production latencies differentiate or interact across conditions, a 2 × 2 repeated-measures ANOVA with factors language context (French vs. German) and Stimulus Type (Words vs. PW) was performed. In addition, a paired *t*-test was performed contrasting PWs in French context versus PWs in German context.

Unless otherwise stated, significance threshold was set at *p* < 0.05. All data analyses were performed using IBM SPSS Statistics 19 (2012).

### ELECTRICAL NEUROIMAGING ANALYSIS

#### ERP waveform analyses

Waveform analyses were performed to determine time periods where ERP amplitude differences occurred between the conditions PWs in French context versus PWs in German context.

Time-frame wise paired *t*-tests were computed between the evoked potentials to the PW read in the French vs. in the German context for each electrode. Only differences lasting at least 11 time frames were retained with an alpha criterion of 0.05.

#### Topographic patterns analyses

A topographic pattern analyses was applied to the ERP to determine whether and when distinct configurations of brain network were engaged in response to the PWs when read in the French vs. German context. This approach is based on evidence that the ERP map topography does not vary randomly across time, but remains quasi-stable over 20–100 ms functional microstates before rapidly switching to other period of stable topography (Lehmann and Skrandies, 1980; Michel et al., 2004; Murray et al., 2008; Britz and Michel, 2011). Spatio-temporal segmentation summarizes ERP data into a limited number of topographical map configurations and identifies time periods during which different conditions evoke different configurations of the electric field at scalp. Because a change in the topography of the scalp-recorded electric field necessarily follows from a change in the configuration of the underlying brain’s active generators, topographic modulations can be directly interpreted as the engagement of distinct brain networks (e.g., Lehmann and Skrandies, 1980).

The most dominant topographic maps appearing in the visual evoked potentials (VEPs) of the group-averaged ERPs from each condition over time were identified with a modified hierarchical cluster analysis, the topographical atomize and agglomerative hierarchical clustering (T-AAHC; Murray et al., 2008). The optimal number of clusters to describe the data set was identified using a modified Krzanowski–Lai criterion (Tibshirani et al., 2001). Then, differences in the pattern of maps observed between conditions in the group-averaged data were statistically tested by comparing the spatial correlation between these template maps from the group-averaged data and each time point of single-subject data from both experimental conditions. For this procedure, referred to as “fitting,” each time point of each ERP from each subject was labeled according to the map with which it best correlated spatially (see Brandeis et al., 1995; Murray et al., 2008). The output of fitting is a measure of relative map presence in milliseconds, which indicates the amount of time over a given interval that each map, which was identified in the group-averaged data, best accounted for the response from a given individual subject and condition. Repeated-measures ANOVA was applied with the factors Condition (PWs in French, PWs in German) and Maps to analyze whether map presence is depending on condition.

The present multivariate topographic analyses have the advantage of being reference-independent (Michel et al., 2001, 2004) and insensitive to pure amplitude modulations across conditions as topographies of normalized maps are compared. Therefore, this approach is not biased by a priori hypotheses about electrode location(s) or period of interests (POIs) at which effects might be expected unlike classical analyses of single-electrode average evoked potentials (Tzovara et al., 2012).

#### Electrical source estimations

Electrical source estimations were calculated using a distributed linear inverse solution and the local autoregressive average (LAURA) regularization approach^1^ (Grave de Peralta et al., 2001, 2004). The results of the above topographic pattern analysis defined the time period over which intracranial sources were estimated and statistically processed. ERPs for each participant and condition (PW in French context versus PW in German context) were first time-averaged over the period showing a significant topographic modulation. Then, intracranial sources were estimated for the resulting one time-sample ERP for each participant and condition and statistically compared at each solution point between the PWs in French context Condition versus PWs in German context condition using paired *t*-tests. The solution space included 3005 nodes, selected from a 6mm × 6mm × 6 mm grid equally distributed within the gray matter of the averaged brain of the Montreal Neurological Institute (MNI; courtesy of Grave de Peralta Menendez and Gonzalez Andino, University Hospital of Geneva, Geneva, Switzerland). In order to control for multiple comparisons, only solutions with a minimal cluster size of 15 consecutive points (*k*_E_) were retained (see also De Lucia et al., 2010; Knebel and Murray, 2012). Significance threshold was set at *p* < 0.05.

## RESULTS

### BEHAVIORAL RESULTS

Mean accuracy (SD) on the whole group of 12 subjects were for Words in French context 98% (7%), words in German context 98.5% (8%), PWs in French context 93% (4%) and PWs in German context 93% (3%). For words in French context, 0% intrusion, 0.23% (2.43%) orthographic, 01.06% (6.47%) phonological, 0.38% (2.34%) intonation and 0.16 % (2.49%) other errors were observed. For PWs in French context, 3.8% (4.09%) intrusion, 1.88% (2.13%) orthographic, 1.03% (1.23%) phonological, 0.48% (0.76%) intonation and 0 % other errors were observed. For words in German context, 0% intrusion, 0.38% (3.24%) orthographic, 0.35% (3.30%) phonological, 0.59% (5.16%) intonation and 0.16 % (2.49%) other errors were observed. For PWs in German context, 3.4% (3.15%) intrusion, 2.8% (1.75%) orthographic, 0.13% (0.37%) phonological, 0.3% (0.47%) intonation, and 0.13 % (0.37%) other errors were observed.

Repeated-measures ANOVA with factors language context (French vs. German) and Stimulus Type (Words vs. PW) was performed to investigate whether accuracy rates differentiate or interact across conditions. This analysis revealed no main effect of language context [*F*(1,11) = 0.85, *p* = 0.378, $\eta_{p}^{2}$ = 0.071], a main effect of Stimulus Type [*F*(1,11) = 33.09, *p* < 0.001, $\eta_{p}^{2}$ = 0.751] and no interaction between language context and Stimulus Type [*F*(1,11) = 0.12, *p* = 0.741, $\eta_{p}^{2}$ = 0.010]. Paired *t*-test showed no difference in accuracy rates between PW in French context and PW in German context [*t*(11) = 0.61, *p* = 0.553, $\eta_{p}^{2}$ = 0.33).

Repeated-measures MANOVA with language context (French, German) as independent and Language Intrusion Errors, Orthographic Errors and Phonological Errors as dependent variables was performed to investigate whether error types in PW reading differentiate across language contexts. This analysis revealed a significant multivariate effect for language context [*F*(3,11) = 6.28, *p* = 0.010, Wilk’s λ = 0.369, $\eta_{p}^{2}$ = 0.631]. *Post hoc* univariate tests showed that the depended variable “Phonological Errors” significantly differentiated across French and German language contexts (French > German; *F*(1,13) = 13.30, *p* = 0.003, $\eta_{p}^{2}$ = 0.506). No statistical difference was found across language contexts for the dependent variables “Language Intrusion Errors” [*F*(1,13) = 0.10, *p* = 0.759, $\eta_{p}^{2}$ = 0.008] and “Orthographic Errors” [*F*(1,13) = 3.72, *p* = 0.076, $\eta_{p}^{2}$ = 0.223].

Mean RTs (SD) on the whole group of 12 subjects were for words in French context 720 ms (168 ms), Words in German context 706 ms (165 ms), PWs in French context 730 ms (172 ms) and PWs in German context 718 ms (164 ms).

Repeated-measures ANOVA with factors language context (French vs. German) and Stimulus Type (Words vs. PW) was performed to investigate whether production latencies differentiate or interact across conditions. This analysis revealed no main effect of language context [*F*(1,11) = 2.59, *p* = 0.136, $\eta_{p}^{2}$ = 0.191), no main effect of stimulus type [*F*(1,11) = 0.02, *p* = 0.883, $\eta_{p}^{2}$ = 0.002] and no interaction between language context and stimulus type [*F*(1,11) = 0.96, *p* = 0.349, $\eta_{p}^{2}$ = 0.080]. Paired *t*-test showed no difference between PW in French context and PW in German context [*t*(11) = 1.58, *p* = 0.143, $\eta_{p}^{2}$ = 0.03].

### ELECTRICAL NEUROIMAGING RESULTS

#### ERP waveforms

**FIGURE 2:**
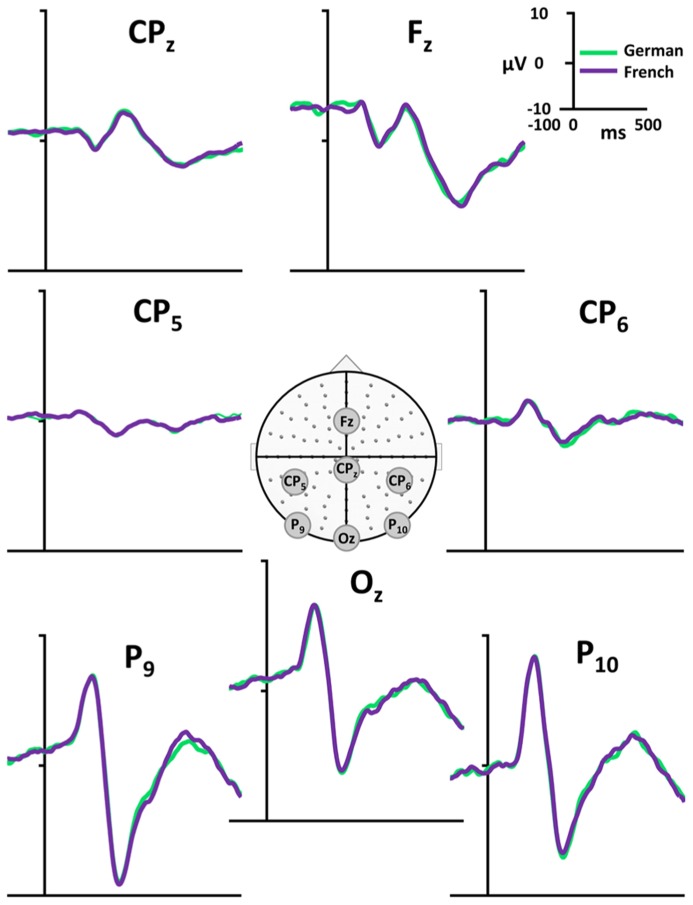
**Exemplar ERP waveforms.** Exemplar group-averaged ERP waveforms (F_z_, CP_z_, Cp_5_, CP_6_, P_9_, P_10_, O_z_) to PW reading in French (violet) and German (green) language context are plotted in microvolts as a function of time. In the middle of the figure, the array of the 128 electrodes with the electrode position of the displayed waveforms is presented.

**FIGURE 3:**
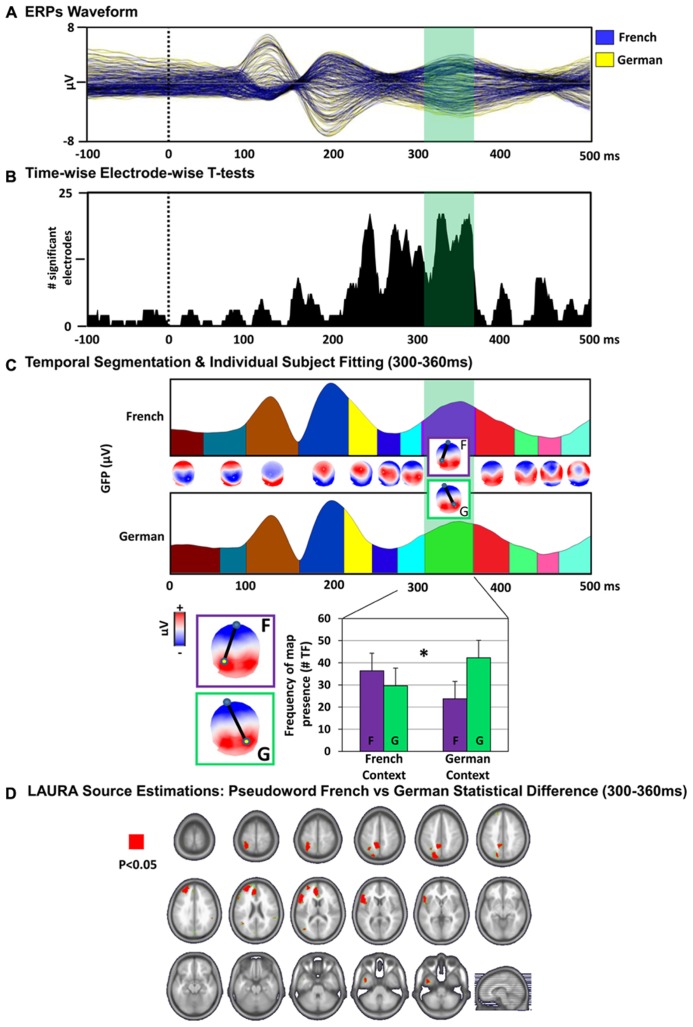
**Electrical neuroimaging results.** **(A)** ERPs waveform. The group-averaged ERPs to PW reading in the French (blue) and German (yellow) language context are displayed in microvolts as a function of time relative to stimulus onset (dotted black line). The time period showing significant (*p* < 0.05) topographic differences between the conditions is indicated in green. **(B)** Time-wise electrode-wise *t*-tests. Results of the time-wise paired *t*-tests at each of the 128 scalp electrodes from the group-averaged ERP waveforms are shown (*p* < 0.05). **(C)** Topographic pattern analysis. ***Top***: Topographic pattern analyses identified 12 time periods of stable electric field topography across the collective 500 ms post-stimulus period form the group-averaged ERPs. Topographies (i.e., maps) are shown with the nasion upward and left scalp leftward. The dipole represents the positive and negative maximum of the electric field topography measured at the scalp. Two distinct maps were identified for one of these time periods (300–360 ms) for PWs in the French context (map “F”) versus German context (map “G”) conditions. ***Bottom: ***The reliability of this observation at the group-averaged level was assessed at the single-subject level using a spatial correlation fitting procedure. The relative map presence in time frames (TF) of each template map provides a measure for the amount of time a given template map, which was identified in the group-averaged data, is present in an individual subject and condition (see “Material and Methods”). Over the 300–360 ms post-stimulus period, the map “F” characterized more frequently the response to the PWs in the French context and the map “G” in the German context condition. There was a significant interaction between language context condition and map presence over the 300–360 ms period [*F*(1,13) = 5.91, *p* = 0.03]. Error bars indicate SEM. **(D)** Distributed LAURA source estimations. Paired *t*-tests were performed for each of the 3005 solution points for time-averaged ERPs over the period of topographic modulation (300–360 ms after stimulus onset), revealing differential (*p* < 0.05) activation of the left inferior frontal gyrus (German > French), left superior parietal gyrus (French > German) and left anterior cinguar cortex (German > French) when reading the PWs in the French versus the German language context.

Evoked potential waveforms to the PWs presented in the two language context are depicted in **Figure 2** for seven exemplar electrodes and in **Figure 3A** for all 128 electrodes.

Paired *t*-tests between the ERP to the PWs in the French context versus in the German context revealed an increase in the number of electrodes showing a statistically significant difference over the time interval of 220–360 ms post-stimulus onset [*p* < 0.05, >1 ms, **Figure 3B**].

#### Topographic pattern analysis

Agglomerative hierarchical clustering was applied on the ERPs to identify the pattern of predominating topographic maps of the electric field at the scalp in the cumulative group-averaged data. The output of the topographic pattern analysis is displayed in **Figure 3C**. The global explained variance of the T-AAHC analysis was 97%. The topographic pattern analysis identified the same sequence of stable topographic maps for group-averaged ERPs from the French context and German context condition, except for the 300–360 ms post-stimulus onset time period. Over this period, different maps were observed for the PW in French context versus German context conditions. The reliability of this observation at the group-average level was assessed at the single-subject level using a spatial correlation fitting procedure (see “Material and Methods”). The individual-subject fitting revealed a significant interaction between language context condition and map over the 300–360 ms period [*F*(1,13) = 5.91, *p* = 0.03]. The map “F” characterized more frequently the response to the PW in the French context and the map “G” in the German context condition, indicating the engagement of distinct configurations of intracranial generators in PW reading across language context in this time window.

#### Electrical source estimations

In order to localize the effect in the brain space, paired *t*-tests of LAURA distributed source estimations between PWs in French context and PWs German context condition were performed for each of the 3005 solution points for time-averaged ERPs over the POI defined by the topographic pattern analysis (300–360 ms post-stimulus). This analysis revealed a significant difference of activation within the left inferior frontal gyrus (German > French; *p* < 0.05; *k*_E_ = 15), left superior parietal areas (French > German; *p* < 0.05; *k*_E_ = 15) and left anterior cingulum (German > French; *p* < 0.05; *k*_E_ = 15; **Figure 3D**).

## DISCUSSION

We investigated the spatio-temporal impact of orthographic depth on reading. Identical PWs were presented to highly proficient bilinguals embedded either in a deep orthographic (French) or in a shallow orthographic (German) language context. The lexical context in which the stimuli were presented (80% words and 20% PWs) has been designed to force initial automatic word reading in the pre-activated context and to force PW reading in the corresponding orthographic depth. Our results show that orthographic depth induced by language context indeed impacts brain response to reading physically identical stimuli. The topography of the ERPs to identical PWs differed 300–360 ms post-stimulus onset when the PWs were read in different orthographic depth context, indicating distinct brain networks engaged in reading during this time window. Analysis of electrical source estimation over the period of topographic modulation showed a differential engagement in left inferior-frontal, left superior parietal and left anterior cingular areas in the deep versus shallow condition.

### TIMING OF THE EFFECT OF ORTHOGRAPHIC DEPTH

The topography of the ERPs to identical PWs differed around 330 ms post-stimulus onset when the PWs were read in different orthographic depth context. Because distinct topographies necessarily follow from distinct configuration of the underlying brain network (e.g., Lehmann and Skrandies, 1980), our result indicates that the language context modulates the brain networks involved in reading. Because subject-related factors (Proficiency, Age of Acquisition, Immersion) were controlled across language context and only the orthographic depth of reading physically identical PWs was modulated, the topographic differences most likely reflect an adaptation of the reading processes to the orthographic depth of the language being read.

The 330 ms latency of the topographic modulation has been associated to processing stages involved in grapheme to phoneme conversion in previous studies (Bentin et al., 1999; Huang et al., 2004; Proverbio et al., 2004; Simon et al., 2004, 2006; Grainger et al., 2006; Hauk et al., 2006; Ashby et al., 2009; Carreiras et al., 2009). This period precedes the semantic processing previously found to take place around 450 ms (Bentin et al., 1999; Simon et al., 2006) and is subsequent to letter identification occurring around 200 ms (Maurer et al., 2005; Brem et al., 2006; Martin et al., 2006; Appelbaum et al., 2009; Lin et al., 2011).

**FIGURE 4:**
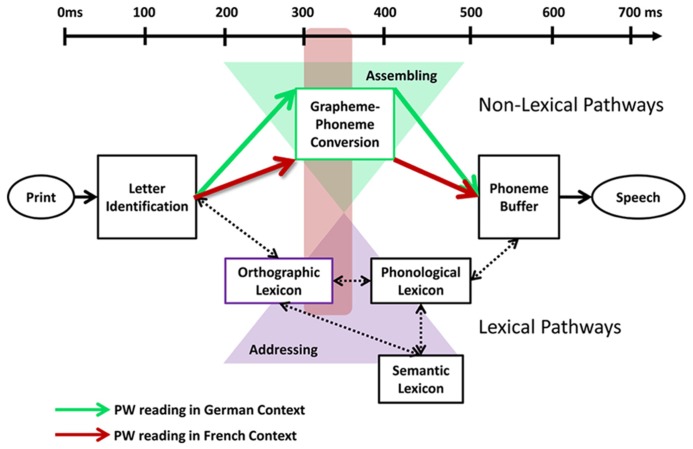
**The orthographic depth hypothesis by Katz and Feldman (1983) posits that different reading routes are engaged depending on the type of grapheme/phoneme correspondence of the language being read.** Shallow orthographies with consistent grapheme/phoneme correspondences favor encoding via non-lexical pathways (assembled reading; green triangle), where each phoneme is sequentially mapped to its corresponding grapheme. In contrast, deep orthographies with inconsistent grapheme/phoneme correspondences favor lexical pathways (addressed reading; violet triangle), where phonemes are retrieved from memory structures. With regard to this framework, we propose that the topographic effects 300–360 ms after stimulus onset (red square) reflect a modulation of the routine non-lexical pathways in PW reading by the stronger recruitment of lexical pathways in the deep than shallow language context. Reading in a shallow context activates the non-lexical pathways more strongly than reading in the deep context, which reinforces the non-lexical processing routinely recruited in pseudoword (PW) reading (green arrows). In contrast, reading in a deep context activates the lexical pathways more strongly than the shallow context, which reduces the engagement of the non-lexical pathways routinely recruited in PW reading (red arrows).

The dual route cascade model posits a lexical and a non-lexical route among which graphemes and phonemes are being mapped (Coltheart et al., 2001).On the non-lexical route, each grapheme is sequentially mapped to its corresponding phoneme. The non-lexical route does not rely on lexico-semantic representations and is thus preferentially recruited in regular, non- and pseudo-words (e.g., Jobard et al., 2003). In contrast, on the lexical route, phonemes are retrieved from memory, i.e., from orthographic and phonological lexical representations. The lexical route is efficient for encoding words, especially irregular words, in which phonological codes do not follow simple grapheme-phoneme rules (e.g., Jobard et al., 2003). Thus, whereas PWs predominately follow the non-lexical route, words may follow either of these routes. The orthographic depth hypothesis (Katz and Feldman, 1983; Katz and Frost, 1992) assumes that for words the predominant engagement of each route depends on the orthographic regularity of a language. In transparent orthographies, non-lexical pathways are preferentially activated to map graphemes and phonemes. In contrast, the sequential mapping on the non-lexical pathways does not fit grapheme to phoneme mapping in languages with irregular orthographies. Instead, irregular languages favor lexical pathways and phonemes are retrieved from memory structures. With regard to this framework, we propose that the topographic effects reflect a modulation of the engagement of the routine non-lexical route in PW reading across language context. Furthermore, we assume that the routine non-lexical route in PW reading was likely modulated by the variable manipulated in the present design, namely the orthographic depth of language contexts (i.e., word reading). We suggest that, when reading words across French and German language contexts, the modulation of orthographic depth in French versus German words may lead to different engagement of one or the other reading route. This modulation of reading routes across language contexts in word reading due to differences in orthographic depth might impact the routine non-lexical pathways recruited in PW processing. Thus, the topographic modulation found in PW reading might be explained by the fact that reading (German) words in a shallow context activates predominantly non-lexical pathways, which reinforce the non-lexical processing routinely recruited in PW reading in the shallow versus deep context. In contrast, reading (French) words in the deep context may activate predominantly lexical pathways, which reduce the engagement of the non-lexical pathways routinely recruited in PW reading in the deep versus the shallow context. Thus, the topographic modulation at 330 ms might reflect a modulation of non-lexical processing in PW reading due to a modulation of reading routes by the orthographic depth of language contexts (**Figure 4**).

Alternatively, one might consider that the modulation of reading route selection by orthographic depth was not restricted to word reading, but directly impacted PW processing. Thus, PW reading might have recruited non-lexical pathways in the shallow and lexical pathways in the deep context. However, the use of low-lexical target stimuli (PWs) and the absence of a differential engagement of lexico-semantic networks across conditions in the results of electrical source estimation over the period of topographic modulation (see “Location of the effect of orthographic depth”) speak against a direct and in favor of an indirect modulation of reading route by orthographic depth. Nevertheless, further research is needed to unravel the precise mechanism underlying the orthographic-related reading route modulation.

Given the fact that the 300–350 time window has also been associated to (early) semantic processing in reading (Proverbio et al., 2008; Yum et al., 2011), an alternative account of our results would thus be that lexical pathways, when reading in the deep orthographic context, induced stronger attempt of semantic processing of PWs (Holcomb et al., 2002; Deacon et al., 2004; Carreiras et al., 2007; Vergara-Martinez et al., 2013) compared to reading in the shallow orthographic context. This assumption is supported by the relative late latency of the effect found in the present study, which may rather reflect a modulation of semantic access than grapheme to phoneme conversion across language context. Indeed, studies on phonological processing have suggested latencies around 200 ms to be critically engaged in grapheme to phoneme conversion (Sereno et al., 1998; Proverbio and Zani, 2003; Wheat et al., 2010). In contrast, the few studies focusing on a modulation of orthographic depth consistently reported latencies around 300 ms to be linked to grapheme to phoneme conversion (Simon et al., 2006; Bar-Kochva and Breznitz, 2012). Thus, with the present design enabling isolating the orthographic depth, later latencies could have been expected. In addition, the high number of significant electrodes differentiating across language contexts found in the time-wise electro-wise *t*-test may indicate that the impact of orthographic depth on PW reading was subliminally initiated earlier (around 220 ms; **Figure 3B**). However, several reasons speak against a modulation of semantic processing by orthographic depth independent of the timing of the effect. First, semantic effects were likely reduced by the utilization of PWs (Friedrich et al., 2008). Second, the task did not strengthen semantic processing as participants had only to read aloud the stimuli. Third, neighborhood size, indicating how many (real) words can be created from a PW by changing one letter without changing letter position, was low and balanced across language context. Fourth, a potential semantic meaning due to a resemblance of a PW to a real word should lead to prolonged but not shortened semantic processing and thus unlikely be measured at an early latency (<400 ms) but rather at a late latency compared to words (>450 ms; Coch and Mitra, 2010). Finally, our results of electrical source estimation underlying the topographic effects confirm the absence of an engagement of semantic networks which have anatomically been linked to inferior and middle temporal and inferior parietal areas (Price, 2000).

Our results thus suggest that distinct brain networks support PW reading 300–360 ms post-stimulus onset when they were read in different orthographic depth context. We propose that these distinct brain networks reflect a modulation of the non-lexical grapheme to phoneme conversion routinely engaged in PW reading by the activation of different reading routes in word reading across language contexts. More precisely, reading (German) words in a shallow context may preferentially activate non-lexical pathways, which strengthen the engagement of the non-lexical pathways routinely recruited in PW reading in the shallow versus the deep context. In contrast, reading (French) words in a deep orthographic context may preferentially recruit lexical pathways, which reduce the reliance on routinely recruited non-lexical pathways in PW reading in the deep versus the shallow context. Thus, the topographic modulation in PW reading might indirectly reflect the engagement of different reading routes across the orthographic depth of language contexts.

### LOCATION OF THE EFFECT OF ORTHOGRAPHIC DEPTH

Statistical analyses of electrical source estimations over the period of topographic modulation support the hypothesis of orthographic-related reading route modulation by showing differential engagement in the deep versus shallow conditions in left inferior-frontal, left superior parietal and left anterior cingular areas.

The left inferior frontal region (part of Broca’s area complex) was activated stronger when reading in the German than French context. The inferior frontal activation might indicate enhanced engagement of phonological processing when reading PWs in the shallow orthography in contrast to reading in the deep orthography. Previous findings showed that inferior frontal regions are involved in grapheme to phoneme conversion (Fiebach et al., 2002; Heim et al., 2005; Rodriguez-Fornells et al., 2006; Wheat et al., 2010) and in enhanced short term memory capacities of non-lexical pathways compared to lexical pathways (Jobard et al., 2003; Nixon et al., 2004).

However, our findings contrast with the results by Paulesu et al. (2000) for an enhanced engagement of Broca’s area in the deep (English) versus the shallow (Italian) language, suggesting an enhanced involvement of inferior frontal regions in lexical than non-lexical processing. The contradictory findings on the engagement of inferior frontal regions in reading route processing may originate from the functional distinction of Broca’s subunits. While the anterior part [Brodmann area (BA) 45] has been associated to lexical processing, the posterior part (BA 44) has been linked to play a crucial role in grapheme to phoneme conversion (Fiebach et al., 2002; Heim et al., 2005). Here, the low spatial resolution of inverse solutions restricts an attribution of the source estimation to the anterior or posterior region within the inferior frontal area. However, we consider our results to most likely reflect enhanced engagement of phonological processing in the posterior inferior frontal lobe (BA 44) when reading PWs in the shallow orthography in contrast to reading in the deep orthography. The stronger engagement of phonological processes when reading in the German than the French context may indicate that the bilingual reader relies more strongly on the non-lexical route, because unlike in the French context, the non-lexical route is strengthened by both, the type of stimuli (PW) and orthographic depth of language context (shallow). Thus, the stronger activation of phonological inferior frontal regions when reading in the shallow than the deep orthographic context may indicate enhanced engagement of phonological non-lexical pathways.

Additionally, inferior frontal regions have been associated to the motor control of speech articulators (Wheat et al., 2010). One alternative explanation of our findings may thus be that language context modulated motor planning. However, previous literature indicates that motor preparation, i.e., phonetic encoding, in reading starts later, namely after (approximately) 350 ms (Moller et al., 2007; Laganaro et al., 2013). Thus, the differential engagement of inferior frontal regions across orthographic depth seems more likely to reflect a modulation of phonological than motor planning processing.

A more pronounced engagement of non-lexical networks could have been expected, as numerous reading studies have demonstrated broad networks to critically underlie grapho-phonological processing covering temporal, parietal and frontal brain regions (Jobard et al., 2003; Ischebeck et al., 2004). The absence of an anatomically broadly distributed difference in activation in non-lexical networks across language contexts might be related to the fact that the present paradigm contrasted PW versus PW reading. In contrast, most studies investigating anatomical correlates of lexical and non-lexical reading routes compared words and PWs (Jobard et al., 2003). In word versus PW contrasts, the extensive differences in network activation found may be related to differences between the stimuli (lexicality, familiarity and/or physical form). In contrast, the present PW versus PW design may reveal networks related specifically to the variable manipulated, i.e., the orthographic depth of PW reading. Consequently, spatially restricted networks might be expected to show differential activity compared to those found in classical studies on reading routes contrasting word versus PW.

The differential engagement of parietal–cingular areas may follow from a modulation of attentional demands across language context.

The activity within superior parietal areas (BA 7) was stronger when reading in the French than German context. In reading, superior parietal areas have been advanced to contribute to visual attention, which could be involved in modifying the reading strategy (Rosazza et al., 2009; Lobier et al., 2012). This interpretation is in line with our hypothesis assuming a modulation of the non-lexical route in PW reading by the stronger recruitment of lexical pathways in the deep than the shallow language context. According to this hypothesis, the stronger engagement of parietal areas might reflect enhanced visual attention related to the recruitment of less routine non-lexical pathways strengthened by PW reading in the deep versus shallow orthographic context. This conclusion is supported by the results of reading error analysis, which revealed significantly more phonological errors when reading the PWs in the deep (French) versus shallow (German) language context (among comparable overall accuracy rates). Thus, the recruitment of less routine non-lexical pathways may have increased phonological inaccuracy when reading the PWs in the deep versus shallow context.

However, alternative explanations could account for the effect found, as parietal areas have been put forward to be involved in a variety of cognitive tasks, including eye movements (especially IPS; Chen et al., 2013; Zaretskaya et al., 2013) spatial orientation (Cabeza and Nyberg, 2000) and multimodal integration (Macaluso et al., 2003). Even though many variables were controlled in the present design (e.g., short stimuli to avoid eye movements) or not required to perform the task (e.g., multimodal integration), the lack of an a priori hypothesis formulated on the parietal engagement and the complexity of functions attributed to this region limit the credibility of our conclusion.

The anterior cingular activity (parts of BA 24, BA 32, and BA 33) was stronger for reading in the German than French context. The cingulate cortex has been linked to inhibitory control and error detection (Garavan et al., 2002; Ridderinkhof et al., 2004), but to our knowledge, its role in reading is currently unknown. In addition to the lack of literature, the lack of an a priori hypothesis formulated on the engagement of cingular regions in the present study prevents us from drawing reliable conclusions. Further research is required to elucidate its role in reading processing.

Cingular activities have been found in proficiency-related control processes (Abutalebi, 2008; Magezi et al., 2012). In the current study, the modulations of activity in the anterior cingulum could be due to a higher proficiency in French than German. However, the experimental setting consisted of separated language contexts, which prevented ongoing language selection, and in turn minimized proficiency-related effects (Abutalebi, 2008). Behavioral results further showed that production latencies did not differ across language context, indicating balanced attentional load across languages. Finally, the scores of the computer-based as well as the self-evaluated proficiency assessments did not differ across languages. Together, the differential engagement of cingular areas unlikely reflects proficiency-related control processes. Instead, we consider these effects to be directly related to the modulation of reading routes by orthographic depth.

The temporal dynamic of the responses to PWs in a German versus French context also support our hypothesis for a modulation of reading route by orthographic depth. Given the timing of the effect between early (letter identification) and later (semantic) processing and our design enabling isolating the effect of orthographic depth, the topographic difference are likely to reflect different networks engaged in grapheme to phoneme conversion across language context 330 ms post-stimulus onset. We propose that the engagement of the non-lexical reading route routinely involved in PW reading is modulated by the activation of distinct reading routes in word reading across language context. Reading (German) words in a shallow context may activate non-lexical processing, which reinforces the involvement of the non-lexical pathways routinely recruited in PW reading, reflected in a stronger engagement of frontal phonological areas in the shallow versus the deep orthographic context. In contrast, reading (French) words in a deep orthographic context may weaken the non-lexical pathways routinely recruited in PW reading. The recruitment of less routine non-lexical pathways in PW reading might be reflected in a stronger engagement of visuo-attentional parietal areas in the deep versus shallow orthographic context.

Since in the present paradigm, many (real) words were used to create language context, the additional inclusion of words into the analyses may have helped to disentangle the nature of the effects found. However, we think that the joint analysis of words and PWs would unlikely help clarifying the interpretation of the present results because of the following reason: To compare words and PWs (e.g., in terms of pathways engaged), an interaction would be needed between the factors stimulus type (words, PWs) and orthographic depth (shallow, deep; Nieuwenhuis et al., 2011). However, when designing the experiment, the words were not selected to be included in the analysis, but to induce strong language context and to ensure identical performances of natural bilingual reading across languages. As a result, there are important differences in physical proprieties, semantic categories, letter frequency distribution or familiarity of the word stimuli across languages. These differences cannot be controlled a posteriori [or for some of them even a priori (familiarity)]. The effect of the confounding factors in the word stimuli would impact the results of a 2 × 2 design and could not be disentangled. Thus the result of a 2 × 2 analyses of our data could not be interpreted.

Several limitations of the current study constrain the interpretability of our results. First, investigating the language system in bilinguals might be less straightforward compared to investigating monolinguals, due to the two languages cohabiting in the brain. This complexity is majorly linked to switching between languages and inhibition of one language (Golestani, 2014). The results of our study, investigating low-level pre-semantic processing, should thus unlikely be affected by higher control strategies related to language switching or selection/inhibition. Moreover, the task was performed in separated language context sessions, further minimizing cognitive control strategies (Abutalebi, 2008). Finally, our bilingual within-subject design minimizes socio-cultural effects and other confounds induced by between-group comparison differences in brain activity and increases the statistical sensitivity of our analyses as each subject is compared to itself.

Second, language skills are probably never perfectly matched across languages, even if statistically comparable in our group. Marginal effects can be argued with regard to the language used to perform mental arithmetic’s, the first language spoken by the mother and the language preferred to watch TV. However, we consider these differences to have unlikely impacted reading performance because of the following reasons: the variables showing differences across languages are related to oral language production, in contrast, variables directly linked to the task, i.e., written language skills (reading books, school, computer-based reading evaluation) showed no differences across languages. In addition, behavioral results (equal RTs/accuracy across languages) speak in favor of balanced proficiency and potential effects of proficiency are minimized by the two separated language context session (Abutalebi, 2008). Finally, 22 *t*-tests were performed to compare bilingualism variables across languages. Multiple hypothesis testing enhances the risk of false positive findings (Miller, 1966). Consequently, a correction for multiple comparisons should be applied to counteract false positive findings. In **Table 1**, significance thresholds are depicted as uncorrected, because we wanted to be as conservative as possible. When applying a correction for multiple comparisons [Bonferroni (Dunn, 1961) or Holm-Bonferroni (Holm, 1979)], none of the variables tested reaches significance level.

Third, the use of PWs as target stimuli might have enhanced attentional demands and the differences found might reflect controlled instead of automatic processing. Indeed, response accuracy was lower during PW than word reading, indicating that PW reading might have enhanced cognitive control. However, equal RTs across words and PWs suggest that the inaccurate responses occurred pre-attentively during “automatic” reading. Additionally, equal RTs across stimulus type reflect that the “word-likeliness” of the PWs (unlike letter strings or non-words) and the strong language context (generated by adding four times more words than PWs) possibly facilitated the task. More importantly, the task was the same across conditions, thus control strategies should unlikely explain the results. Further, equal RTs and accuracy of PW reading across conditions support a comparable engagement of cognitive control processes, which should thus be cancelled out in the analysis. Finally, the effects found around 300 ms are unlikely to reflect higher processing mechanisms such as cognitive control strategies (Chouiter et al., 2013). Nevertheless, it is important to note that the use of PWs as target stimuli likely reinforced assembled/non-lexical reading in both languages and may thus not reflect natural everyday reading, especially in the French context.

Fourth, the low spatial resolution of EEG inverse solution limits the interpretability of the spatial aspects of our data. However, the high-density EEG montage (128 channels) enables that the localization accuracy with LAURA is in the order of the grid size, i.e., about 0.6 cm^3^ (Michel et al., 2004). In addition, these limitations were partially remedied by applying statistical parametric mapping analyses to the source estimation (Michel et al., 2004). Even when the estimated activity in brain regions is of unrealistic size, statistical analysis can reveal whether differences between experimental conditions are reliable. Finally, a conservative statistical approach was applied in order to interpret only the most pronounced effects. Nevertheless, our labeling of areas should be interpreted with caution and with respecting these limitations.

Fifth, a further limitation of the study is the small sample size (*n* = 14) which is explained by the application of rigid inclusion criteria in order to have an optimally balanced group of native French-German bilinguals. Small sample sizes usually have low statistical power, which enhances the risk of false negative and false positive findings (Button et al., 2013). To estimate the statistical power of our study, a compromised *post hoc* power analysis was performed (Erdfelder, 1984) using G^*^Power Software (Faul et al., 2009) which resulted, assuming a medium effect size (Cohen’s *d* = 0 0.5), an alpha level of 0.05, a ratio q = beta/alpha = 1, and a sample size of 14 matched pairs, in a power (1- beta error) of 75%, which can be labeled as medium to large power size.

Sixth, due to differences in brain representations of language processing between bi- and monolinguals (Hernandez et al., 2000, 2005; Mechelli et al., 2004; Rodriguez-Fornells et al., 2005, 2006; Kovelman et al., 2008) a generalization of our results obtained by investigating highly proficient bilinguals to non-native bi- or monolinguals is limited. In addition, only female subjects participated in the study, further limiting a generalization to male populations. It would be interesting for future research to investigate the manipulation of reading route by orthographic depth in male and mixed populations.

Finally, the dual route cascade model, in its original form, may be too rigid as a template to project our results. Instead, our results should be discussed in terms of a parallel engagement of both routes, but one may be predominantly activated compared to the other depending on the orthographic regularity of the language.

## CONCLUSION

The present study reveals insights into the neural underpinnings of orthographic regularity processing. Our findings complement current literature on reading processing and support the orthographic depth hypothesis (Katz and Feldman, 1983), by showing that not only the lexicality/familiarity of a stimulus, but also its orthographic regularity may modulate the engagement of reading routes.

## Conflict of Interest Statement

The authors declare that the research was conducted in the absence of any commercial or financial relationships that could be construed as a potential conflict of interest.

## Footnotes

1 LAURA inverse solution is a weighted minimum norm method together with the LAURA regularization approach. The LAURA method calculates a current density value at each solution point. The local auto-regressive average regularization approach describes the spatial gradient across neighboring solution points (Grave de Peralta etal., 2004; Michel etal., 2004). Specifically, the strength of the source regresses with distance according to electromagnetic laws (i.e., the square root of the distance).
